# Angiosarcoma after radiotherapy: a cohort study of 332 163 Finnish cancer patients

**DOI:** 10.1038/sj.bjc.6603805

**Published:** 2007-05-22

**Authors:** A Virtanen, E Pukkala, A Auvinen

**Affiliations:** 1 Tampere School of Public Health, FI-33014 University of Tampere, Tampere, Finland; 2 Finnish Cancer Registry, Institute for Statistical and Epidemiological Cancer Research, FI-00170 Helsinki, Finland; 3 Finnish Cancer Institute, FI-00170 Helsinki, Finland; 4 Research and Environmental Surveillance, STUK – Radiation and Nuclear Safety Authority, FI-00881 Helsinki, Finland

**Keywords:** hemangiosarcoma, cohort studies, radiation effects, radiotherapy, sarcoma

## Abstract

We evaluated the risk of angiosarcoma after radiotherapy among all patients with cancers of breast, cervix uteri, corpus uteri, lung, ovary, prostate, or rectum, and lymphoma diagnosed in Finland during 1953–2003, identified from the Finnish Cancer Registry. Only angiosarcomas of the trunk were considered, this being the target of radiotherapy for the first cancer. In the follow-up of 1.8 million person-years at risk, 19 angiosarcomas developed, all after breast and gynaecological cancer. Excess of angiosarcomas over national incidence rates were observed after radiotherapy without chemotherapy (standardised incidence ratio (SIR) 6.0, 95% confidence interval (CI) 2.7–11), after both radiotherapy and chemotherapy (SIR 100, 95% CI 12–360), and after other treatments (SIR 3.6, 95% CI 1.6–7.1). In the regression analysis however, the adjusted rate ratio for radiotherapy was 1.0 (95% CI 0.23–4.4). Although an increased risk of angiosarcoma among cancer patients is evident, especially with breast and gynaecological cancer, the excess does not appear to be strongly related to radiotherapy.

Sarcomas are malignant mesenchymal tumours and angiosarcoma develops from the endothelium of a blood vessel. It is an uncommon cancer type that occurs among men and women, more often among older adults and usually in the skin, soft tissue, breast, or liver (Schoen and Cotran, 1999).

The risk of a second cancer is increased among persons who have had a cancer previously (IARC, 2000), owing both to shared risk factors and treatment for the first cancer, both chemotherapy and radiotherapy increasing cancer risk. In our previous study, radiotherapy was found to be a risk factor for subsequent bone and soft tissue sarcoma (Virtanen *et al*, 2006). Ionising radiation is one of the few known causes of angiosarcoma (Miller *et al*, 1996; Zahm *et al*, 1996; Huang and Mackillop, 2001; Fletcher *et al*, 2002; Yap *et al*, 2002). We have investigated the risk of angiosarcoma in relation to chemotherapy and radiotherapy in a large, population-based cohort of cancer patients.

## MATERIALS AND METHODS

The study cohort included patients with first primary cancers diagnosed during 1953–2003 in the Finnish Cancer Registry database, which has almost complete coverage of cancers in Finland since 1953 (Teppo *et al*, 1994). National Research and Development Centre for Welfare and Health (STAKES) granted the permission to use the Finnish Cancer Registry database.

The cohort included patients with first primary cancers of the breast, cervix uteri, corpus uteri, lung, ovary, prostate, and rectum, as well as Hodgkin's and non-Hodgkin's lymphomas, selected because they are treated both with radiotherapy and other modalities, mainly chemotherapy, surgery, and hormonal therapy. Small-cell carcinoma of the lung was excluded because it is rarely treated with radiation therapy. In addition, radiotherapy for these cancers involves almost exclusively the trunk, which allowed restriction of the end point and hence more specific evaluation of the effect, by exclusion of angiosarcomas in the head and extremities, that is, outside the radiation beam. The selected cancers are relatively common and constitute 47% of all the registered cancers in men and 50% in women in 1953–2003 in Finland (Finnish Cancer Registry, 2005).

The analyses were stratified by type, treatment, cancer site, and age at diagnosis (10-year age group) of the first cancer, sex, and length of follow-up time (0, 1–4, 5–9, 10–14, 15, or more years since diagnosis of the first primary cancer). Treatment was classified into four groups: treatment without chemotherapy or radiotherapy (e.g. surgery or hormonal treatment), chemotherapy without radiotherapy, radiotherapy without chemotherapy, and combined chemotherapy and radiotherapy.

The cohort was followed up for subsequent angiosarcomas and lymphangiosarcomas, starting at diagnosis of the first primary cancer and ending at death, emigration, diagnosis of angiosarcoma, or the common closing date (31 December 2003). Information on emigration and vital status was obtained from the Population Register Centre by computerised record linkage.

The outcome event was defined as a histologically confirmed angiosarcoma or lymphangiosarcoma on the trunk, those outside the trunk being excluded, as only radiation treatment to the trunk is relevant to the study. The anatomic sites of the first cancer included oesophagus, stomach, small intestine, large intestine, rectum, biliary ducts, gallbladder, liver, pancreas, lung, mediastinum, breast, cervix uteri, corpus uteri, uterus and other female reproductive organs, ovary, prostate, kidney, bladder, urinary tract, skin of the trunk, bone of the trunk, and soft tissue of the trunk.

Expected numbers were calculated by multiplying the person-years at risk with the national incidence rates stratified by sex, 5-year calendar period, and 5-year age group. The standardised incidence ratio (SIR) was calculated as the ratio of observed to expected numbers of cases. The 95% confidence intervals (CI) were estimated assuming that the observed number followed Poisson distribution and the expected number being constant (Breslow and Day, 1989). As the estimated minimal latency time for radiation-induced solid tumours is 10 years (Boice *et al*, 1996), special focus was given to the follow-up time of 10 or more years after diagnosis of the first primary cancer. To adjust for the effects of age, sex, follow-up time, and first primary cancer type, which may differ between treatment categories, Poisson regression modelling was used (Clayton and Hills, 1993). Cumulative incidence (Satagopan *et al*, 2004) was assessed as an estimate of angiosarcoma risk at 10 years of follow-up. Also, the number needed to harm (Walter, 2000) was evaluated as an estimate of the number of patients who should receive radiotherapy in order to induce one excess sarcoma during 10 years of follow-up. The Stata 8.0 software (Stata Corporation, College Station, TX, USA) was used in the analysis.

## RESULTS

The cohort included a total of 332 163 patients with first primary cancers. Among the cohort members, 54% were women and 76% were aged 55 years or more at the time of diagnosis of the first primary cancer. Mean age was 59 years among patients with radiotherapy and 66 years among patients without radiotherapy. The follow-up yielded 1.79 million person-years at risk, with a mean length of follow-up time of 5.4 years. Of the total person-time at risk, 4% was provided by the patients receiving both radiotherapy and chemotherapy, 41% after radiotherapy alone, and 5% after chemotherapy alone.

In the follow-up, 19 cases of angiosarcoma were observed *vs* 3.8 expected (Table 1), all in women; 12 of the cases were in patients over 55 years at diagnosis of the first cancer. No lymphangiosarcomas were recorded in the follow-up. At 10 years of follow-up, the cumulative incidence, directly standardised by age, for angiosarcoma was 0.02/1000 (95% CI 0.00/1000–0.06/1000) for patients who were not treated with radiotherapy. After radiotherapy with or without chemotherapy, cumulative incidence was 0.06/1000 (95% CI 0.02/1000–0.11/1000), that is, one excess case expected within 10 years among 22 200 patients receiving radiation treatment.

Overall, the risk of angiosarcoma was increased in every treatment group, except after chemotherapy alone, among whom no angiosarcomas were found (Table 1). In the radiotherapy group, nine cases were found, SIR 6.0 (95% CI 2.7–11) for the entire follow-up and SIR 4.1 (95% CI 0.84–12) for the follow-up of 10 or more years. Risk was higher for patients treated with both radiotherapy and chemotherapy, SIR 100 (95% CI 2.5–560, two cases) for the follow-up after 10 years, when it was also increased among patients not treated with radiotherapy or chemotherapy. Among women after radiotherapy with or without chemotherapy, risk was increased (SIR 5.3, 95% CI 1.5–14, four cases) for follow-up of 10 years or more (Table 2). After 10 years of follow-up, an excess risk was also found among patients not treated with radiotherapy, SIR 7.7 (95% 2.1–20, four cases). No consistent pattern of risk in relation to age at observation was observed.

The angiosarcomas occurred among patients with cancers of the breast, cervix uteri, corpus uteri, and ovary, the majority of them (14 observed cases *vs* 1.7 expected) being among breast cancer patients (Table 2). Among the latter treated with radiotherapy with or without chemotherapy, a significant excess risk was found, SIR 11 (95% CI 4.8–20) for the entire follow-up time.

In the regression analysis, the crude rate ratio was 0.94 (95% CI 0.34–2.6) for the radiotherapy group, compared to those not so treated, and was not materially affected by adjustment for age, first cancer type, and follow-up time (RR 1.0 95% CI 0.23–4.4). A separate regression analysis for breast cancer patients gave an adjusted RR of 1.0 (95% CI 0.23–4.4) for radiotherapy. The effect of treatment did not differ significantly by age, follow-up time, or site of the first cancer type (*P*>0.1).

## DISCUSSION

Our results indicate that our cancer patients have a five-fold elevated risk of angiosarcoma compared with the rest of the population. All cases were in women and were confined to patients with breast or gynaecological cancers. We did not find a strong relationship with radiotherapy, but our findings are consistent with an increased risk related to combined radiotherapy and chemotherapy. However, despite the large cohort with more than 330 000 patients with first primary cancer, the number of angiosarcomas was small.

The results of this study are consistent with the few previous studies of angiosarcoma after radiotherapy. In a SEER data study of second malignancies among 270 000 breast cancer patients, angiosarcomas were found to comprise 56.8% of the sarcomas occurring in the field of prior radiation (Yap *et al*, 2002). In a population-based retrospective cohort study of 195 000 breast carcinoma patients, an increased risk of soft tissue sarcoma was found, especially for angiosarcoma: SIR 26.2 (95% CI 16.5–41.4) in the radiotherapy cohort with an increased relative risk (RR 15.9, 95% CI 6.6–38.1) compared to those not so treated (Huang and Mackillop, 2001); the SIRs were lower in our study, with no excess after radiotherapy compared with other treatments.

Post-irradiation sarcomas have mainly followed breast and gynaecologic cancers (Kim *et al*, 1998), breast cancer, lymphoma, and carcinoma of cervix (Brady *et al*, 1992), and cancers of breast and female reproductive organs (Wiklund *et al*, 1991). In our study, the angiosarcoma cases occurred following breast cancer and gynaecologic cancers, with no cases among men. This suggests an association between female hormonal factors and angiosarcoma.

In previous studies, the minimal latency for radiation to cause a solid tumour has been estimated as 10 years (IARC, 2000). In a cohort study of breast cancer patients, the RR of angiosarcoma after radiotherapy reached its maximum during 5–10 years of follow-up (Huang and Mackillop, 2001). Our study indicates an increased angiosarcoma risk after 10 years both after radiotherapy and other treatments. The cumulative incidence at 10 years indicated an increased risk related to radiotherapy, not observed in other analyses, owing to an early excess (within 10 years of treatment).

Patient or disease characteristics other than treatment may confound the results, if they affect angiosarcoma risk. Several factors influence selection for radiotherapy (Easson, 1991). Features such as detailed treatment scheme, tumour location, or histology were not evaluated in this study and further case–control analyses are needed.

We were unable to determine whether the angiosarcoma of the trunk occurred within the irradiated volume, or to provide measures of risk related to doses to specific organs, but only estimates of the overall risk of angiosarcoma.

By basing our study on comparisons of patients with similar first cancers and different forms of treatment, the effect of aetiologic factors underlying the first cancer were largely eliminated as a possible cause of the angiosarcoma. Another strength of this study was the large, representative study population, covering practically all eligible Finnish cancer patients diagnosed during 1953–2003, while record-linkage with the population registry allowed complete follow-up.

These results indicate an increased risk of angiosarcoma among cancer patients, especially those with breast and gynaecological cancers. Similar increased risks were found both among patients treated with radiotherapy and those without radiotherapy. Further analyses focusing more closely on the irradiated tissue and dose–response are needed.

## Figures and Tables

**Table 1:** Observed and expected numbers of angiosarcomas on the trunk and SIR by follow-up time and treatment for the first primary cancer 1953–2003

	**Entire follow-up**		**Follow-up of 10 or more years**	
**Treatment**	**O/E**	**SIR (95% CI)**	**O/E**	**SIR (95% CI)**
Chemotherapy	0/0.07	0.0 (0–53)	0/0.02	0.0 (0–180)
Radiotherapy	9/1.5	6.0 (2.7–11)	3/0.74	4.1 (0.84–12)
Both	2/0.02	100 (12–360)	1/0.01	100 (2.5–560)
Neither	8/2.2	3.6 (1.6–7.1)	4/0.61	6.6 (1.8–17)
Total	19/3.8	5.0 (3.0–7.8)	8/1.4	5.8 (2.5–11)

CI=confidence interval; E=expected; O=observed; SIR=standardised incidence ratios.

**Table 2:** Observed and expected numbers of angiosarcomas and SIR for breast and other cancers by follow-up time, and radiation treatment for the first primary cancer 1953–2003

		**Entire follow-up**		**Follow-up of 10 or more years**	
**First primary cancer**	**Radiotherapy**	**O/E**	**SIR (95% CI)**	**O/E**	**SIR (95% CI)**
Breast	Yes	9/0.85	11 (4.8–20)	2/0.36	5.6 (0.67–20)
	No	5/0.84	6.0 (1.9–14)	1/0.28	3.6 (0.09–20)
	Total	14/1.7	8.3 (4.5–14)	3/0.64	4.7 (1.0–14)
					
Other^a^	Yes	2 /0.61	3.3 (0.40–12)	2/0.39	5.1 (0.62–18)
	No	3/0.56	5.4 (1.1–16)	3/0.24	13 (2.6–37)
	Total	5/1.2	4.3 (1.4–9.9)	5/0.63	7.9 (2.6–18)
					
Total	Yes	11/1.5	7.5 (3.8–13)	4/0.75	5.3 (1.5–14)
	No	8/1.4	5.7 (2.4–11)	4/0.52	7.7 (2.1–20)
	Total	19/2.9	6.6 (4.0–10)	8/1.3	6.3 (2.7–12)

CI=confidence interval; E=expected; O=observed; SIR=standardised incidence ratios.

a After cancers of cervix uteri (one case with and one without radiotherapy), corpus uteri (two without radiotherapy), and ovary (one with radiotherapy).
